# Strategies discovery in the active allothetic place avoidance task

**DOI:** 10.1038/s41598-022-16374-1

**Published:** 2022-07-25

**Authors:** Avgoustinos Vouros, Tiago V. Gehring, Bartosz Jura, Małgorzata J. Węsierska, Daniel K. Wójcik, Eleni Vasilaki

**Affiliations:** 1 Department of Computer Science, The University of Sheffield, Sheffield, S1 4DP UK; 2 Faculty of Management and Social Communication, Jagiellonian University, 30-348 Cracow, Poland; 3 Nencki Institute of Experimental Biology of Polish Academy of Sciences, Warsaw, 02-093 Poland; 4 Institute of Neuroinformatics, University of Zurich and ETH Zurich, Zurich, Switzerland

**Keywords:** Behavioural methods, Spatial memory, Computational science

## Abstract

The Active Allothetic Place Avoidance task is an alternative setup to Morris Water Maze that allows studying spatial memory in a dynamic world in the presence of conflicting information. In this task, a rat, freely moving on a rotating circular arena, has to avoid a sector defined within the room frame where shocks are presented. While for Morris Water Maze several studies have identified animal strategies which specifically affect performance, there were no such studies for the Active Allothetic Place Avoidance task. Using standard machine learning methods, we were able to reveal for the first time, to the best of our knowledge, explainable strategies that the animals employ in this task and demonstrate that they can provide a high-level interpretation for performance differences between an animal group treated with silver nanoparticles (AgNPs) and the control group.

## Introduction

Navigation in a stable environment is based on allothetic or idiothetic memory or both. However, these two kinds of memory are brought into conflict when relevant and irrelevant (misleading) information is presented simultaneously^1,2^. Formation of proper allothetic memory in such conditions requires segregation of information that involves cognitive coordination processes^3,4^. The Active Allothetic Place Avoidance (AAPA) task, also known as the Carousel maze^5–8^, is an experimental paradigm to study functioning of allothetic memory in the presence of conflicting information. In this test, animals are placed on a dry circular arena where they can freely walk. In such conditions, they have to learn to avoid a shock sector, which is not marked physically but is fixed regarding the distal, relevant cues from the room, in the presence of misleading proximal cues from the arena. The entrance to this sector is signalled by an application of a short-lasting mild electric shock to the rat’s paws, which is repeated at short time intervals until the rat leaves this sector. Thus, proper navigation in the AAPA task requires on-going active segregation of irrelevant local cues (e.g. faeces, urine) from the arena and use of only the distal relevant cues from the room. The AAPA task is a variation of the passive place avoidance task^9,10^, in which animals are placed in a chamber divided into two compartments, dark and light. Here, they also need to avoid shocks, which are presented in the dark compartment, but contrary to the AAPA task, they do so by suppressing their activity and remaining in the light compartment.

The performance in the AAPA task has been shown to be strongly hippocampal-dependent^11^ and more sensitive to its unilateral blockade^3,7^, than the performance in the Morris Water Maze (MWM)^12,13^, which is a commonly used navigation task in which animals are placed in a pool of water and have to find a hidden platform to escape the water. The difference may follow from the fact that in the MWM only distal cues are available and useful for animals to orient themselves^14^. Another advantage of the AAPA task, compared to the MWM, is that swimming is less natural for the rat than freely moving on the stable ground of the arena.

Commonly used measures to assess memory in the AAPA task^15–18^ are: the total number of entrances to the shock sector, the number of shocks received, the time to the first entrance, and the maximal time of avoidance (the total path length and linearity of the path are considered here as measures of locomotor activity, not memory). Although very useful, these performance measures of memory do not give a direct indication about how the animals behave during the acquisition of memory and how their behaviour changes within and between sessions. In the case of spatial memory testing in the Morris Water Maze, the limitation of single performance measures has been identified long time ago^19,20^. The individual measures alone, like time or distance to the platform, simply cannot account for the variety of different behaviours or strategies observed in the experiments. Therefore, other analysis methods based on the classification of the swimming paths of the animals have been proposed over the years^21,22^. These methods combine a number of different measures of trajectories to define a set of classes of behaviour. In^23,24^ a more fine-grained method for classifying the MWM trajectories was presented. In that study multiple overlapping segments of the swimming paths, instead of the complete paths, were classified. This made it possible to identify changes of exploration strategy within a single trial and to highlight subtle behavioural differences between groups of tested animals where other methods failed.

Here we show for the first time (to the best of our knowledge) that a similar methodology to^23,24^ can be applied in the AAPA task to help to identify strategies. Using the original record of the rats moving in the AAPA task we develop an analysis method for the AAPA experiments that is complementary to standard performance measures and which can give further insight into how the behaviour of animals changes over time and differs between groups of animals. As a case study and to validate the method, a set of AAPA experiments investigating how silver nanoparticles (AgNPs) coated with bovine serum albumin (AgNPs(BSA)) affect the spatial memory of rats^25^ is analysed here with the proposed method and compared against the standard approach.

It is important to understand the effects of silver nanoparticles (AgNPs) on higher brain functions such as memory and consciousness. Increasing use of silver nanoparticles (AgNPs) as nanomaterials in industry and in medicine due to their antibacterial, antiviral and antifungal properties results in increased human exposure to silver^26–28^. Toxicological *in vivo* and *in vitro* studies indicated that AgNPs are able to cross brain-blood barrier and are a risk factor for the brain^28^. Recently, Wȩsierska et. al^25^ evaluated the effects of orally administered AgNPs (BSA), in comparison to non-treated control, on spatial memory, which engage cognitive coordination processes for on-going stimuli segregation. It was shown that oral exposure to a low dose AgNPs (BSA) induces detrimental effect on memory and cognitive coordination processes. Moreover, silver ions rather than AgNPs were found particularly in the hippocampus, a major structure for higher brain functions. We use this established case to validate the proposed method of analysis, we show the results are consistent with the previous findings but also provide additional insight and allow better quantification of the studied behaviour.

The method developed here, as in the case of the MWM swimming paths, is based on analysing trajectory segments instead of the full trajectories. Trajectories are therefore first split into segments which are grouped into different clusters with the help of a clustering algorithm. However, contrary to the analysis method for the MWM trajectories^23,24^, we use supervised rather than semi-supervised methods, with labels corresponding to the clusters that have been identified via the clustering process. The proposed analysis method is first introduced and then applied to a data set from AAPA experiments to demonstrate that it can be successfully used to identify different types of behaviour. The observed differences in behaviour between treated and untreated animals are then compared with standard analysis results. It is shown that both give consistent results but that our approach provides complementary information about animal behaviour not apparent from individual measurements alone. As such it can be used as a biomarker of specific impairments to differentiate strains or treatments.

## Results

We propose an analysis method for the active allothetic place avoidance experiments which focuses on identifying stereotypical behavioural patterns of animals. Our method is based on segmenting the trajectories of the animals and then grouping similar segments by features such as their position in relation to the sector where shocks were applied, geometry, and movement speed, among others. The segmentation was done in two steps. At the first step, the start point of a segment was defined as the first data point after exiting the shock sector; the end point was defined as the last data point before next entrance to the shock sector. In the second segmentation step (called subsegmentation) the start point was defined as first data point at which the local angular speed crossed the threshold, set by the median value of angular speed (defined as a given number of rad/sec). The end point was defined as the last data point before which the local angular speed again crossed the threshold value. The obtained trajectory fragments are subsequently called subsegments. Full details of the segmentation procedure are provided in the Methods section.

We applied the method to a set of experimental data acquired at the Nencki Institute of Experimental Biology, Warsaw (^25^; see Accession codes below for the link to the original data). In the experiments 20 rats were submitted to the AAPA task and their trajectories were recorded. Of those animals, 10 were administered orally with silver nanoparticles AgNPs (BSA) (30 mg/kg); the other 10, which received water, were the untreated control group. Each animal was submitted to 5 sessions of 20 minutes each during which they could move freely in a circular rotating arena and had to learn to avoid a shock sector which was fixed with respect to the room cues. This was followed by one 20 min test session (memory retrieval test) in which the shocks were not given, otherwise the sector was treated as in the other sessions. Effective performance of place avoidance indicates proper spatial memory functioning.

Previous performance analysis methods of the AAPAT typically rely on comparisons of individual performance measures, which can well reflect how successfully each group of animals avoided the shock sector, but offer little insight into the differences in their detailed behaviour. The method we introduce here, on the other hand, is able to identify and highlight the differences in behavioural patterns of the studied animals. This not only leads to a better understanding of how animals learn to navigate within the arena and avoid based on the room cues, but also makes it possible to identify differences in behaviour between the different treated groups as well as during memory acquisition and retrieval, which would otherwise not be possible.

### Standard performance measures

**Figure 1:**
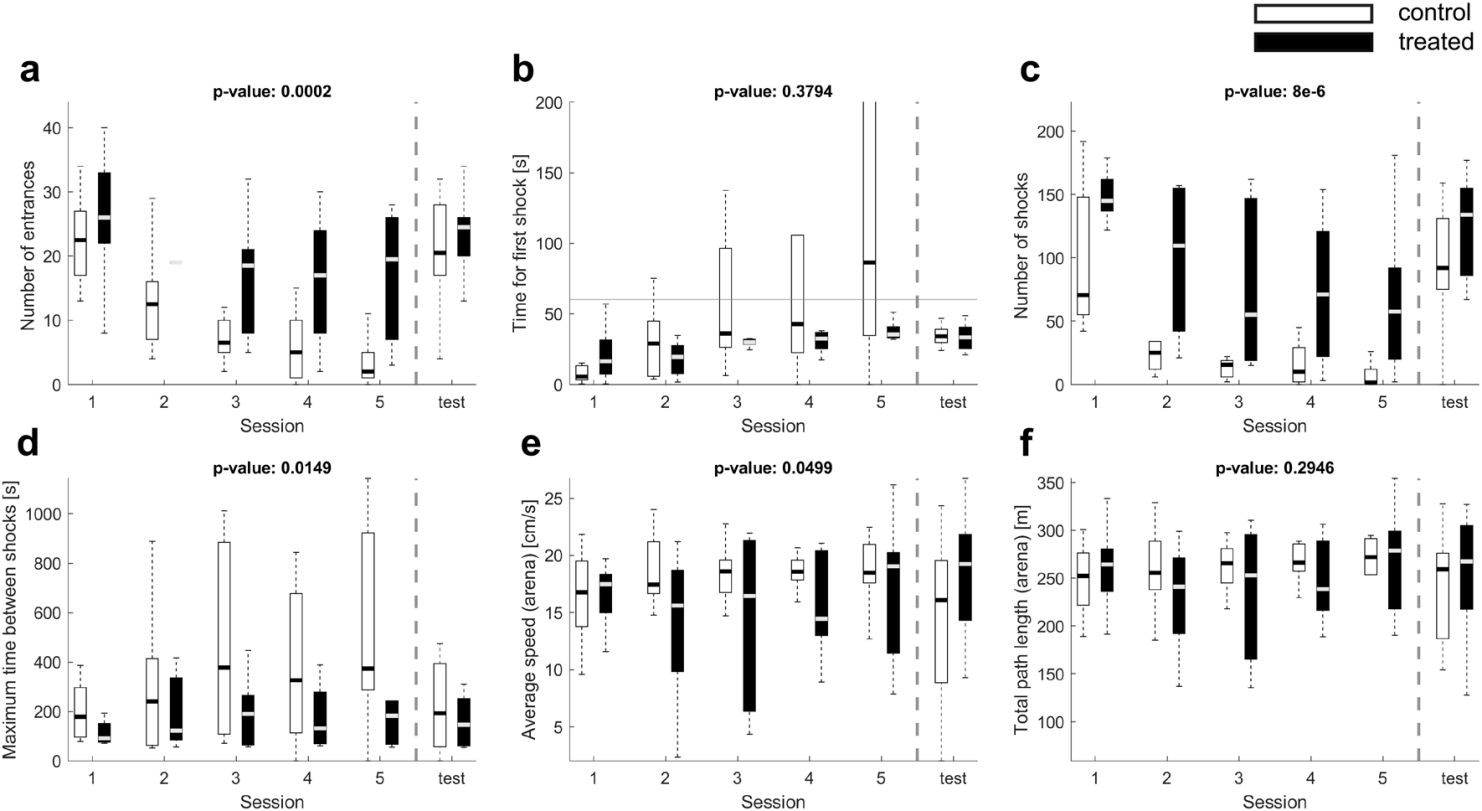
Comparison of performance between untreated control (white) and treated (black) animals over a set of 5 sessions followed by 1 session of memory retrieval test. Boxes represent the first, second (median, shown as a band) and third quartiles; whiskers are the minimum and maximum values. Friedman test p-value was used to compare both animal groups during the five experimental sessions. Treated animals perform more entrances to the shock sector than the control animals (number of entrances, **a**), receive more shocks as a result (number of shocks, **c**) and re-enter the shock sector more often than control animals (maximum time avoided, **d**). No significant differences were detected between the groups on the time until they first enter the shock sector (time for first shock, **b**) or the total length of their paths (**f**). There was significant difference between the average speed distributions in both groups (**e**). These results suggest that animals in the control group are able to quickly learn how to avoid the shock sector and perform on average much better than treated animals.

Standard performance measures for the AAPA usually compare the number of entrances to the shock sector, the number of shocks received by an animal during a session, the time to the first shock, and the maximum time avoided (see^18^ for a detailed description of these and other statistics). Figure 1 shows the above performance measures for rats treated orally with silver nanoparticles and the untreated control group during consecutive sessions of spatial memory acquisition (Fig. 1a–d). The data used here were previously described by Węsierska et al.^25^ where full details of the experiments can be found, see Methods for an overview. Figure 1 is shown for reference to make it easier for the reader to follow our arguments. The main difference as compared with the figure in the previous paper is that there we showed results as mean ± SEM. Here we decided to show the results as median with interquartile range, which we feel is more adequate for data which are not normally distributed.

The results show that the treated animals made more entrances, received more shocks with shorter maximum time avoided within a session than the untreated ones. There was no significant difference to the time for first shock between the groups. Contrary to differences in memory measures, no difference between groups was found in locomotor activity measured with the total path length during the whole session, although there was a statistically significant difference in average speed between the groups (Fig. 1e–f). Statistical significance of differences between groups was established with the Friedmann test, also used in our earlier work^23,24^, for the first five sessions which constituted the actual AAPA task.

Although the performance measures shown in Fig. 1 identify a clear difference in performance in the spatial memory task between treated and nontreated animals, they provide no indication about the types of behaviour that lead to such differences in the first place. The new analysis method we propose in what follows, on the other hand, takes a closer look at the motion of the animals and gives a better insight about how the treatment that animals were subjected to affects their behaviour.

Observe that for the number of entrances and the maximum time avoided the distribution of values on the sixth session (“test”, after 4 days’ break) is similar to that on the first session. This is the situation when the rats do not receive shocks in the sector. What we see behaviourally is that they initially remember the presence of sector, but since the pain is not coming, they revert to treating this area as equivalent to others. So the distribution is similar as in the first session although the motivation is different. Keep in mind that on the last session, from the analytical standpoint, all data are equivalent, however, even though “shocks” are being recorded in the data, they are actually not provided to the animal (this part of the system is off). For the time to the first entrance (T1) the results on the test day are noticeably better than on the first day although the difference between groups is gone. For a detailed interpretation of those results see Węsierska et al. 2018^25^.

### Strategies discovery

We applied K-means clustering to identify the strategies that animals use on the AAPA. We revealed 5 strategies that we named taking inspiration from previous work in the experimental procedure of the Morris Water Maze^24^:

1. *thigmotaxis*: the animal is moving around the periphery of the arena.
2. *incursion*: the animal is moving to inner parts of the arena.
3. *focused*: the animal is moving around a particular area of the arena.
4. *chaining*: the animal is mainly passively transported within the inner parts of the arena till the shock sector.
5. *avoidance*: the animal moves away from the shock sector moments before it receives a shock.

**Figure 2:**
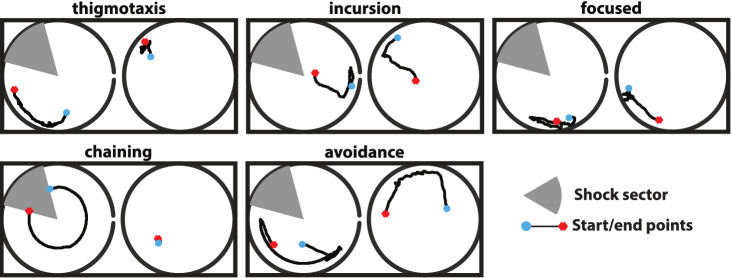
The five stereotypical behaviours, manually identified. Left plots: room coordinates; right plots: arena coordinates.

The strategies are shown in Fig. 2.

### Classification of trajectory motifs

**Figure 3:**
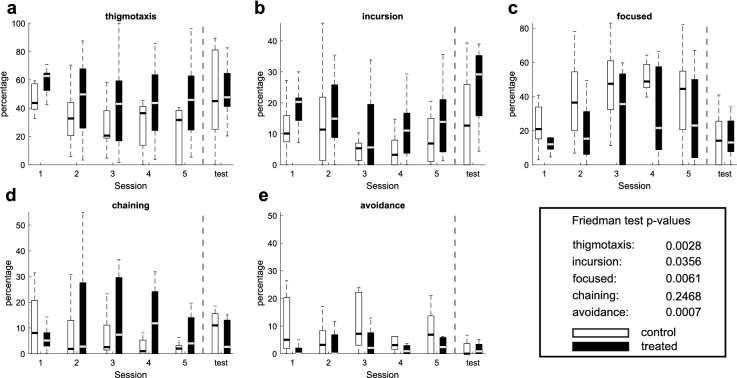
Percentage of subsegments falling under each behaviour for the treated (black) and control (white) animal groups over each session. All the animals were tested for a set of 5 sessions followed by one memory retrieval test. The Friedman test p-value (shown in the legend) was used to compare both animal groups during the five experimental sessions. According to the plots there are significant differences among the two groups in the behaviours: thigmotaxis (**a**) and incursion (**b**) in favour of the treated group (*p*-value equal to 0.0028 and 0.0356) and avoidance (**e**) and focused (**c**) in favour of the control group (*p*-values equal to 0.0007 and 0.0061). These results suggest that the control animals attempt to distance themselves from the arena periphery and remain in a specific area of the arena (focused) which supports proper task performance (avoidance), while the treated animals move on periphery (thigmotaxis) or move into the inner part of the arena (incursion). This behaviour did not allow effective avoidance of the sector to-be-avoided, which would indicate that rats from treated group were unable to remember the location of the shock sector and to avoid it when they were close to it. This is consistent with the avoidance efficiency measurements in Fig. 1 (number of entries, number of shocks, maximum time avoided) which indicate impaired avoidance in treated animals compared to the controls. No significant difference was found for chaining (**d**), which is passive transportation of sitting animals by the arena.

We used standard supervised methods to classify data from the two subgroups into the five stereotypical behaviours and compared their differences in using these strategies during the AAPA task, see Fig. 3 and Methods. The choice of specific strategies differed between the groups which indicates differences in the assessment of the location of the to-be-avoided place required for proper performance in the AAPA task. The analysis revealed significant differences in thigmotaxis, incursion, focused, and avoidance strategies. Percentage of subsegments falling under each behaviour for the treated (black) and control (white) animal groups over each session is shown in Fig. 3. All animals were tested for a set of 5 sessions followed by one memory retrieval test. The Friedman test p-value (shown in the legend of Fig. 3) was used to compare both animal groups during the five experimental sessions.

The first strategy involved the movement of rats at the edge of the platform (Fig. 3a). It is natural for rats to avoid the open area and prefer walking near walls. This behaviour of rats has been called thigmotaxis and has been accepted as a consequence of anxiety in response to open space. The opposite natural phenomenon in animals is the gradual reduction of anxiety and exploration of open space, which results in reduction of thigmotaxis. In our study, a reduction of thigmotaxis across days was observed in control rats. In contrast, rats from the AgNPs (BSA) group showed a strong thigmotaxis during training days. Strong thigmotaxis limited the ability to assess the size of the shock sector and as a result, weakened the animals performance in the avoidance task. Using the Friedman test we found a significant difference between the two groups ($p=0.0028$).

Another strategy, which we call ’focused’ indicates the tendency of the rats to move around a specific area of the arena (in the room coordinates or with respect to the shock delivery segment) in a way that did not interfere with the shock delivery segment. This behaviour was conducive to the execution of the avoidance task. Treated rats showed limited ability to use the focused strategy. There was a significant difference between the two groups ($p=0.0061$). The opposite strategy to ’focused’ is the movement of rats into the arena, which we called ’incursion’. Rats using the incursion strategy approached the centre of the arena where there was a high risk of entry to the place to-be-avoided, and a high risk of getting a shock. This strategy was significantly more common in the rats of the treated group ($p=0.0356$). The avoidance strategy, which consisted in keeping a certain distance from the segment with shocks, guaranteed the effective avoidance of shocks. This strategy was used more commonly by the control rats ($p=0.0007$).

In each of the training groups, there were rats that behaved passively and did not perform the active place avoidance task. Consequently, these animals were passively carried around by the rotating arena. The strategy representing this pseudomovement is called chaining. There was no significant difference in the percentage of chaining segments between groups due to the different behaviour on day one and on the following days. However, the percentage for chaining strategy for control animals decreased and for days 2–5 it was more commonly employed by the treated animals. The Friedman test for data on days 2–5 shows *p* = 0.2683 and for days 3–5, *p* = 0.0742.

The described behavioural strategies explain the differences between groups in the execution of the avoidance task. These behaviours could not be quantified and qualitatively determined based on the standard parameters used previously in AAPA experiments.

## Discussion

We have presented a new analysis method for studying spatial memory using the AAPA task that allows us to identify qualitative and quantitative behavioural differences in groups of animals that have undergone different treatments. The method relies on splitting the recorded trajectories of the animals in the arena, computing a set of features for each resulting trajectory segment, and then using a clustering algorithm to identify similar behavioural patterns in the data. The results of the clustering were visually inspected and each cluster was mapped to a class. This method was inspired by our earlier works^23,24^ on the analysis of the Morris Water Maze experiments. In contrast to our earlier methodology, however, where a semi-supervised classification method was used, here we employed standard supervised classifiers for assigning segments to classes.

We applied this methodology to a data set consisting of the trajectories of 20 animals recorded in the Active Place Avoidance Task. Half of the animals were treated with silver nanoparticles, the other half was the untreated control group. Although the standard memory measures show an impairment of spatial memory in the treated animals, the differences in how the active place avoidance task is performed between the two groups become much more evident when their behavioural patterns are compared. Impairment of avoidance in treated animals shows as poor recognition of the position of the shock sector in the room frame coordinates and diminished ability of learning efficient strategies to avoid it. Treated animals show a higher tendency for sitting in one place in the arena until passively entering the shock sector. The new method identified five types of distinct behaviours which, to the best of our knowledge, were never described in the literature before. We named these behaviours after visually similar strategies in MWM.

Unlike MWM, in the allothetic spatial memory task, animals move on a stable ground. Correct completion of the task is not forced by time or distance, as is the case of the Morris water maze. Rats are free to change behaviour during the experimental session. In this context, we expect them to choose the most effective one for the task. Indeed, the identified behavioural strategies provide a verifiable explanation of the observed differences between groups in the avoidance task. We would like to emphasise that these behaviours could not be quantified based on the standard measures used in AAPA experiments. For this, our analysis is a valuable addition to the standard evaluation of data for AAPAT as they enable studying spatial memory in AAPAT in relation to broadly understood strategies for space exploration.

It is believed that the dominant structure for the functioning of declarative memory, including spatial memory, is the hippocampus, whereas the amygdala is mainly responsible for emotional memory. Hence, one might expect a correlation between a certain strategy and the dominant brain structure for a specific memory, e.g. focused or thigmotaxis. The formation of spatial memory is a dynamic process which, apart from changes at the neuronal level, is closely related to time. Observing changes in particular strategies might assist in evaluating behaviour in subsequent stages of memory formation and maintenance.

The method proposed here opens up a new approach to evaluating AAPA experiments. A question that naturally arises is whether the behavioural strategies we observed are universal. Will we observe the same trajectory classes in other AAPA experiments, depending on the treatments and with other strains? Our earlier work on MWM and the strategies identified therein were in fact used to analyse data from different experiments and labs^24,29,30^, suggesting that it might be possible that the same generality holds for the AAPAT. However, we need to highlight a fundamental difference. The strategies in the MWM methodology were taken from the literature, hence from independent datasets. From the variety available, those strategies that seemed more relevant to interpreting our data and to capturing different behaviours were kept. Here, we have chosen the strategies based on the same data on which we performed our analysis, and this might impose limitations. Perhaps a better approach would be to apply the clustering method to various AAPA experiments, reassuring that the selected strategies are found in a variety of data.

Regardless, because of the generality of the strategies detected, we expect that at least the methodology described here can assist in identifying differences in various groups of animals and potentially classify animal dysfunction. An extension of this work might focus on quantifying behavioural changes over time.

## Methods

The data used here were previously described by Węsierska et al.^25^ where full details of the experiments can be found.

### Animals and treatment

Twenty naïve adult (2.5 month-old) male Wistar rats, weighing 270–310 g, were obtained from the breeding colony of The Center of Experimental Medicine of the Medical University of Białystok, Poland. They were accommodated in transparent plastic home cages, four animals per cage, under standard conditions (constant temperature of 22 $^\circ$C, 12:12 light/dark cycle, humidity at 50%). Water and food were available in the cages ad libitum. 28 days before the experiments, ten of the animals were orally administered with 30 mg/kg of body weight (b.w.) of AgNPs in 0.2 mL of saline (experimental group), whereas eight control rats received 0.2 mL saline per rat (untreated control group). All experimental protocols were approved according to the applicable provisions of the national law by the local authority at the Nencki Institute of Experimental Biology, Polish Academy of Sciences, Warsaw, Poland (The First Warsaw Local Ethics Committee for Animal Experimentation (No. 788/2015 of 25.05.20)).

### Experimental setup and protocol

**Figure 4:**
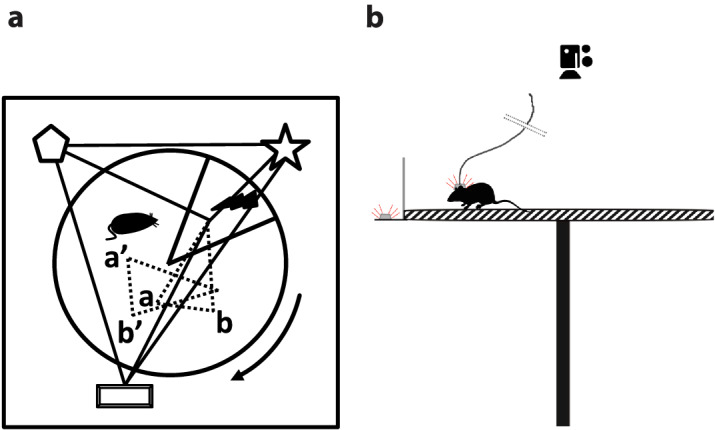
Schematic illustration of the Active Allothetic Place Avoidance task. (**a**) Cues from Room and Arena are present but only the room cues are relevant as the to-be-avoided sector is defined in the room and setup reference frame. Arena-bound misleading cues from the rotating arena must be ignored. The geometrical figures presented outside the arena depict extramaze cues, which provide meaningful reference throughout the experiment. The letters (**a**), (**b**) on the arena depict intramaze cues, which become misleading as the arena rotates. The arrow shows the direction of rotation of the arena. (**b**) Animals are placed on top of an elevated arena which is slowly rotating (1 revolution per minute). They can move freely around the arena but need to learn to avoid the shocks, which are delivered on sector, which is fixed according to the distal room cues. If they enter sector to be avoided, a short-lasting low-current pulse is delivered to their paws and repeated every 1.5 s until they leave this sector. The position of the animals is tracked with two LEDs (one attached to the side of the rotating arena, and one attached to the animal’s head), by a top-mounted camera.

The experiment using setup for the Active Allothetic Place Avoidance task was conducted at the Nencki Institute of Experimental Biology, Warsaw, Poland. The same basic experimental setup as described in^17^ was used. The setup consisted of an aluminium circular arena 80 cm in diameter and a 2 cm rim which rotated with one revolution per minute. The arena was positioned 80 cm over the floor and placed in the centre of a 3x4 meter lightly lit room which contained many stable external visual cues. Infrared light-emitting diodes (LED) for tracking the position of the animals and a 25G (0.50 mm) hypodermic needle electrode were attached to the backs of the rats. A second LED was attached to the periphery of the arena to facilitate calculation of the rat’s position in the reference frame of the arena. The LEDs allowed monitoring the position of the rat by the infrared TV camera which was connected to a computer system. The experimental setup is shown schematically in Fig. 4.

Five recording sessions of 20 min each with a fixed shock sector (in the room coordinates) were performed over five consecutive days. This was followed by a retrieval test session five days later where the shocks were not active. For the sessions with an active shock sector, animals received a short (0.5 s) constant current pulse whenever they entered a predefined 60$^\circ$ shock sector, which remained fixed across the sessions. The amplitude of the shock pulses varied between 0.2 and 0.5 mA and was determined individually for each animal so that shocks did not make the animal freeze or induce attempts to escape the arena. Shocks were repeated every 1.5 s until the animal left the shock sector. The position of the animal and the current state of the electrode (shock active or not) was recorded at 25 Hz using commercial software (Bio-Signal Group, New York).

The animals were kept in groups of four per cage in the animal house during the whole experiment. The rats from the experimental group were fed orally by gavage, administered with 30 mg/kg (group Ag30, n=10). The control rats (group Ctrl30, n=10) received orally 0.2 mL saline per rat. AgNPs and saline were administered once a day from Monday to Friday for four weeks. The higher dose (clinically relevant exposure) used in this study (30 mg/kg b.w.) is a NOAEL (no observable adverse effect level) for AgNPs after oral exposure^31–33^. According to the dose conversion factor from rat to human, the dose of 30 mg/kg b.w. for rats corresponds to the human dose of 4.86 mg/kg b.w.^34^.

The experiment consisted of 5 phases: (1) a handling period to make animals accustomed with the person performing the experiment, animal housing and handling procedures (3 days); (2) treatment period, when the animals were exposed to nanoparticles (28 days); (3) habituation period to get the animals accustomed to the training procedure in the AAPA task (coinciding with the last 5 days of the treatment period) by placing the rat on the stable arena without shocks for a period of 10 min each day; (4) spatial memory training in the AAPA task (one 20 min session per day, 5 days; the shocks are turned on); (5) retrieval test after a 4-days break to test how the rats will keep the memory (1 day; with shock disconnected).

### Data analysis

#### Segmentation of trajectories

**Figure 5:**
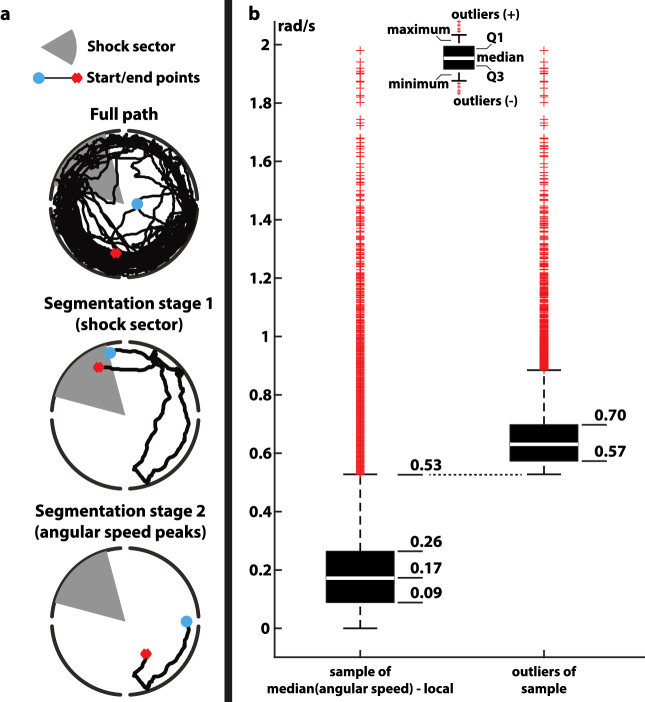
(**a**) Two-step segmentation of the trajectories. In the first step trajectories are split into segments containing only the parts of the paths not falling inside the shock sector. In the second step, sudden changes in the angular speed are taken as the delimiting points of the segments. (**b**) A sample of angular speed changes is taken into consideration and upper outlying values where discarded. These values correspond to sudden changes. Based on this analysis a cut-off value of 0.6 was selected to split the segmented animal paths into subsegments. The cut-off value of 0.5 and 0.55 were also considered for sensitivity analysis.

Our goal was to design a framework allowing us to understand how the strategies of animals for avoiding the shock sector evolve over time (between sessions) and differ between treated and untreated animals. We found it practical to segment the trajectories in two steps. In the first step the recorded trajectories were split into fragments delimited by entrance/exit from the shock sector. That is, only the parts of the trajectories not falling in the shock sector were considered. Specifically, the start point of a segment was defined as the first time/data point after exiting the shock sector; the end point was defined as the last time/data point before the next entrance to the shock sector. Since the length of the trajectories between shocks varied widely, from the order of a few seconds up to the duration of the session (20 min), and since the animals during this long time usually display multiple types of behaviour, these fragments were split further. For an illustration of our segmentation process, refer to Fig. 5a.

The objective of the second segmentation step was to isolate the different behaviours found in a trajectory and to generate a more uniform distribution of segment lengths, to facilitate classification. The second segmentation used the changes in the angular speed as criteria for splitting the trajectory segments. This is because in the Active Allothetic Place Avoidance Test animals have to move in the angular direction in order to evade the shock sector.

This second stage of segmentation (subsegmentation) was performed in the following manner: we processed each segment sequentially point by point ([*time*, *X*, *Y*] coordinate) and for each path subsegment completed after 1 second we computed its median angular speed. The 1 second rule corresponds to subsegments of median length of 2cm. For each additional point added to the subsegment the difference between the median angular speed and the local angular speed is considered; this difference is our criterion if the subsegment should be separated or not and corresponds to peaks of angular speeds. To estimate the cut-off value we considered a sample of approximately 23000 cases of median and local angular speed differences and plotted them in a box plot format (refer to Fig. 5b). In this way we identified that values above 0.53 rad/s correspond to upper outliers. Based on MATLAB’s default box plot function an upper outlier is defined as a value that are greater than $Q3 + 1.5(Q3-Q1)$, where Q1 and Q3 are the 25th and 75th percentile of the values. Such outliers correspond to sharp changes of the angular speed and to select the cut-off value we consider a second box plot, this time using only the outlier values. From the second box plot, we identified that values from 0.57 rad/s up to 0.7 rad/s correspond to Q1 and Q3 respectively thus we selected the value of 0.6 rad/s to be our cut-off value. To allow space for sensitivity analysis for our selection we have also tested the values of 0.5 rad/s and 0.55 rad/s. As a rule of thumb we found that threshold values greater than 0.6 rad/s resulted in longer subsegments falling under more than one category. Subsegments shorter than 5 seconds (median length of 5.4cm) were discarded from further analysis. For an overview of our logic for selecting the cut-off values, refer to Fig. 5b. Statistics of the two segmentation steps can be seen in Table 1.

**Table 1:** Segmentation of trajectories statistics.

Segmentation	Segments	Median length	Min. length	Max. length	Rel. length
(Full paths)	120	24,781 cm	6,095 cm	34,138 cm	100%
1st segmentation	1754	760 cm	51 cm	24,370 cm	88.5%
2nd (cut-off 0.50)	6518	243 cm	2 cm	1394 cm	76.1%
2nd (cut-off 0.55)	6244	271 cm	2 cm	1716 cm	80.8%
2nd (cut-off 0.60)	6041	291 cm	2 cm	1957 cm	84.0%


All lengths are measured in the arena (rotating) reference frame. The last column shows the total length of the resulting segments compared to the input (full paths or segments), i.e., without the short segments or subsegments that were discarded. The figure below the table shows the length distributions of the subsegments for the 2nd stage segmentations.

### Computation of features

For each trajectory segment, a set of 11 features was computed. These features measure different geometrical and positional aspects of the segments, used in the classification (Table 2). Some features are computed using the room reference frame. Other features are computed in the rotating reference frame, i.e., using the real paths swept by the animals on the arena. A detailed description of each feature is given in what follows.

**Table 2:** Features for the data clustering of trajectory segments.

Feature	Unit	Reference frame
Angular distance to shock sector	Rad	Room
Angular dispersion	Rad	Room
Angular dispersion (Arena)	Rad	Arena
Median log radius	–	Room/Arena
IQR log radius	–	Room/Arena
Trajectory centrality	%	Room/Arena
Median speed	cm/s	Arena
IQR speed	cm/s	Arena
Median angular speed	Rad/s	Arena
IQR angular speed	Rad/s	Arena
Speed change frequency	s$^{-1}$	Arena

For a detailed description please refer to the Materials and Methods Section.

#### Angular distance to shock sector

This value measures the angular distance from the centre of the shock sector in the room coordinate frame to the angular centre of the segment. The latter is computed by adding the position vectors of each sample in the trajectory (i.e., the vector to the centre of the arena) and then taking the angle of the resulting vector relative to the middle shock sector angle. If the resulting angle is negative, $2\,\pi$ is added to it so that the resulting values are in the $[0,2\,\pi )$ range.

#### Angular dispersion

The angular dispersion measures the angular spread of the trajectories in the room and arena coordinate frames. It is here defined as the difference between the maximum and minimum angles of the position vectors of all data samples in the trajectory segment.

#### Median/IQR of the log-radius

These values are calculated from the trajectory by computing the distance to the centre of the arena for each data sample, taking the logarithm and then computing the median (interquartile range) of the values. The median and IQR were chosen over the mean and standard deviation because they are less susceptible to outliers.

#### Trajectory centrality

Measures the relative amount of time that the animal spends at the more central regions of the arena. The value is computed by computing the length of the trajectory falling within a concentric circle with a radius of $75\,\%$ of the radius of the arena and dividing this value by the total length of the trajectory.

#### Median/IQR speed

The speed at each trajectory point is computed and the median/interquartile-range of the resulting values is then calculated.

#### Median/IQR angular speed

The angular speed (relative to the centre of the arena) at each trajectory point is computed and the median/interquartile-range of the resulting values is then calculated.

#### Speed change frequency

Measures the number of times that the speed changes abruptly within the segment. Calculated by counting the number of times that the (absolute) speed crosses $25\,\%$ of the median speed of the segment.

### Classes identification

#### Clustering and manual classes identification

To identify classes of behaviour, we used K-Means clustering^35^. More specifically, before the clustering process, we used the MATLAB Principal Component Analysis (PCA) implementation^36^ to reduce the dimensionality of our original dataset. We found that the 8 principal components explain the variability of the data as follows: The first PC explains $82.91\%$ of the variability, the second PC $11.95\%$, the 3rd PC $2.53\%$, the 4th PC $2.04\%$ and the 5th to 8th PC $0.07\%$, $0.03\%$, and $0.01\%$ correspondingly. We applied K-Means clustering on these transformed data and the original dataset, varying the number of clusters from 1 to 10. As a clustering algorithm, we used Lloyd’s K-Means^37^ initialised with the Density K-Means++ method^38^ which worked well in our previous benchmark^39^. We used the R package *clustree*^40^ to visualize a K-Means clustering tree (number of target clusters 1 to 10) for data transformations with different numbers of PCs (2 to 11, leading to different data dimensionality, see also Supplementary Material). We confirmed that our K-Means clustering remained stable among the initial data set and transformed data using a number of PCs greater or equal to 8. Afterwards, we inspected the K-Means trees by visualising samples of subsegments close to the cluster centres. We found that for 5 clusters, we could identify representative, interpretable classes consistent across different numbers of principal components (PCs 2 to 8), see Fig. 2. We then manually assigned the data points for the segmentation with a 0.6 threshold, producing the distributions shown in the main text.

#### Subsegmentation, sensitivity analysis and supervised classification

In addition to 0.6, we performed the 2nd stage segmentation for the threshold values of 0.5 and 0.55. In varying the threshold, we wish to demonstrate that our results do not critically depend on an exact threshold at 0.6 but remain consistent when using similar threshold values. We used supervised classification methods since (i) we have a classification problem at hand and (ii) we avoid the manual procedure followed for the 0.6-threshold. MATLAB’s Statistics and Machine Learning Toolbox^36^ has several classification procedures. We trained each one by giving as input manually classified subsegments obtained by the subsegmentantion with a threshold of 0.6. We estimated the accuracy of each classifier using 10-fold cross-validation. Table 3 lists the tested classifiers. We then consider only the classifiers with accuracy equal to more than $75\%$. We observed that the similarities of these classifiers were in the range of ($80\%$-$85\%$), and by merging them, we could improve the classification performance. More formally, we take the classification result of the classifiers, and we perform classification boosting with majority voting^41^: for each subsegment, we count the number of times that the classifiers agree on each class, and we assign the subsegment to the winning class. In case of a draw, we marked the subsegment as unidentified. We highlight that, to increase the classification performance using the boosting technique of majority voting, the classifiers need to have both high accuracy and also to be diverse^42^. We then used the boosted classifier to classify the data obtained for threshold values of 0.5 and 0.55. We hypothesised that this slight variation would not affect the segments. We should get similar results from manual and automatic classifications if our assumption is correct. Visual inspection of sub-samples seemed to justify the hypothesis. Besides improving the performance, we used the boosted classifier to detect data points with ambiguous classification (draw). We found that $7\%$ of the subsegments remained unidentified; these subsegments were then manually labelled. Our majority voting method boosted the classification performance at $81\%$ accuracy, while the highest single classifier performance was $78.3\%$. The conclusions from this classification process for the subsegmentations for 0.5 and 0.55 threshold values (see Supplementary Material) match the findings for 0.6 (main text). An alternative approach might have been to provide labelled data for threshold values of 0.5 and 0.55, building different classifiers for each and comparing the final results. It would have certainly added to the robustness of the results; however, when performing these experiments, we considered it unnecessary due to the visual similarity of the data produced by the three thresholds and the similarities in their distributions, see Table 1. We emphasise that we base the conclusions drawn on the manual classification of the 0.6 thresholds.

**Table 3:** Performance of classifiers [%].

Classifier	10-fold	F1	Classifier	10-fold	F1
Quadratic discriminant	64.2	0.607	RUSBoost Trees	71.7	0.698
Gaussian Naive Bayes	64.3	0.615	Cosine KNN	72.1	0.713
Subspace discriminant	65.9	0.618	Medium KNN	72.4	0.716
Linear discriminant	66.8	0.632	Coarse Gaussian SVM	72.5	0.717
Fine KNN	67.0	0.659	Weighted KNN	73.2	0.720
Coarse tree	68.7	0.660	Medium Tree	73.5	0.725
Kernel Naive Bayes	69.8	0.662	**Fine Tree**	**75.3**	**0.745**
Coarse KNN	70.4	0.670	**Boosted Trees**	**75.7**	**0.747**
Subspace KNN	70.7	0.684	**Medium Gaussian SVM**	**77.3**	**0.755**
Fine Gaussian SVM	71.3	0.691	**Cubic SVM**	**77.6**	**0.758**
Cubic KNN	71.4	0.692	**Bagged Trees**	**78.1**	**0.764**
Linear SVM	71.7	0.695	**Quadratic SVM**	**78.3**	**0.765**

Classifiers used in majority voting are in [bold].

#### Statistics

Multi-factor testing of variance was done using the Friedman test^43^, a nonparametric test that is well suited for data that is not normally distributed. In our case, the same animals were analysed over multiple sessions, which showed a gradual change in behaviour over time. The p-values shown in our analyses answer the question: if the effect of different treatments (untreated control vs. silver nanoparticles treatment) is identical, what is the chance that a random sampling would result in the distribution of values as far apart as observed? Small p-values (< 0.05 in our analyses) lead us to discard the null hypothesis that the results are identical and the differences are only due to random sampling.

## Supplementary Information

Supplementary Information.

## Data Availability

MATLAB code for the analysis performed in this article is provided at: https://github.com/RodentDataAnalytics/code-aapa. The original data are available at https://repod.icm.edu.pl/dataset.xhtml?persistentId=doi:10.18150/repod.6640789.

## References

[1] Bures J (1997). Dissociation of exteroceptive and idiothetic orientation cues: effect on hippocampal place cells and place navigation. Philos. Trans. R. Soc. Lond. Ser. B Biol. Sci..

[2] Fenton AA, Wesierska M, Kaminsky Y, Bures J (1998). Both here and there: simultaneous expression of autonomousspatial memories in rats. Proc. Natl. Acad. Sci. U. S. A..

[3] Wesierska M, Dockery C, Fenton AA (2005). Beyond memory, navigation, and inhibition: Behavioral evidence for hippocampus-dependent cognitive coordination in the rat. J. Neurosci..

[4] Phillips WA, Silverstein SM (2003). Convergence of biological and psychological perspectives on cognitive coordination in schizophrenia. Behav. Brain Sci..

[5] Stuchlík, A. *et al.* Place avoidance tasks as tools in the behavioral neuroscience of learning and memory. *Physiol. Res.* **62** (2013). 10.33549/physiolres.932635 24329689

[6] Dockery CA, Wesierska MJ (2010). A spatial paradigm, the allothetic place avoidance alternation task, for testing visuospatial working memory and skill learning in rats. J. Neurosci. Methods.

[7] Cimadevilla JM, Wesierska M, Fenton AA, Bures J (2001). Inactivating one hippocampus impairs avoidance of a stable room-defined place during dissociation of arena cues from room cues by rotation of the arena. Proc. Natl. Acad. Sci. U.S.A..

[8] Stuchlik, A., Kubik, S., Vlcek, K. & Vales, K. Spatial navigation: implications for animal models, drug development and human studies. *Physiol. Res./ Acad. Sci. Bohemoslov.* **63** Suppl 1, S237–S249 (2014). 10.33549/physiolres.932660 24564663

[9] Bammer G (1982). Pharmacological investigations of neurotransmitter involvement in passive avoidance responding: A review and some new results. Neurosci. Biobehav. Rev..

[10] Haroutunian, V., Barnes, E. & Davis, K. L. Cholinergie modulation of memory in rats. *Psychopharmacology* 266–271 (1985). 10.1007/BF00432705 3001803

[11] Cimadevilla JM, Kaminsky Y, Fenton A, Bures J (2000). Passive and active place avoidance as a tool of spatial memory research in rats. J. Neurosci. Methods.

[12] Vorhees C, Williams M (2006). Morris water maze: procedures for assessing spatial and related forms of learning and memory. Nat. Protoc..

[13] Morris RG, Garrud P, Rawlins JN, O’Keefe J (1982). Place navigation impaired in rats with hippocampal lesions. Nature.

[14] Morris R (1981). Spatial localization does not require the presence of local cues. Learn. Motiv..

[15] Stuchlik A, Rehakova L, Rambousek L, Svoboda J, Vales K (2007). Manipulation of D2 receptors with quinpirole and sulpiride affects locomotor activity before spatial behavior of rats in an active place avoidance task. Neurosci. Res..

[16] Stuchlik A, Vales K (2008). Role of alpha1- and alpha2-adrenoceptors in the regulation of locomotion and spatial behavior in the active place avoidance task: A dose-response study. Neurosci. Lett..

[17] Wesierska M, Adamska I, Malinowska M (2009). Retrosplenial cortex lesion affected segregation of spatial information in place avoidance task in the rat. Neurobiol. Learn. Mem..

[18] Wesierska MJ, Duda W, Dockery CA (2013). Low-dose memantine-induced working memory improvement in the allothetic place avoidance alternation task (APAAT) in young adult male rats. Front. Behav. Neurosci..

[19] Gallagher, M., Burwell, R. & Burchinal, M. Severity of spatial learning impairment in aging: development of a learning index for performance in the Morris water maze. *Behav. Neurosci.* (1993). 10.1037//0735-7044.107.4.618 8397866

[20] Dalm S, Grootendorst J, de Kloet E R, Oitzl M S (2000). Quantification of swim patterns in the Morris water maze. Behav. Res. Methods Instrum. Comput. J. Psychon. Soc. Inc.

[21] Wolfer D, Lipp H (2000). Dissecting the behaviour of transgenic mice: is it the mutation, the genetic background, or the environment?. Exp. Physiol..

[22] Graziano A, Petrosini L, Bartoletti A (2003). Automatic recognition of explorative strategies in the Morris water maze. J. Neurosci. Methods.

[23] Gehring TV, Luksys G, Sandi C, Vasilaki E (2015). Detailed classification of swimming paths in the Morris Water Maze: multiple strategies within one trial. Sci. Rep..

[24] Vouros A (2018). A generalised framework for detailed classification of swimming paths inside the morris water maze. Sci. Rep..

[25] Wȩsierska M (2018). Silver ions are responsible for memory impairment induced by oraladministration of silver nanoparticles. Toxicol. Lett..

[26] Akter M (2018). A systematic review on silver nanoparticles-induced cytotoxicity: Physicochemical properties and perspectives. J. Adv. Res..

[27] Bondarenko O (2013). Toxicity of ag, cuo and zno nanoparticles to selected environmentally relevant test organisms and mammalian cells in vitro: a critical review. Arch. Toxicol..

[28] Teleanu DM, Chircov C, Grumezescu AM, Volceanov A, Teleanu RI (2018). Impact of nanoparticles on brain health: An up to date overview. J. Clin. Med..

[29] Nizinska K (2021). Behavioral characteristics as potential biomarkers of the development and phenotype of epilepsy in a rat model of temporal lobe epilepsy. Sci. Rep..

[30] Huzard D (2020). Constitutive differences in glucocorticoid responsiveness are related to divergent spatial information processing abilities. Stress.

[31] Jeong GN (2010). Histochemical study of intestinal mucins after administration of silver nanoparticles in sprague-dawley rats. Arch. Toxicol..

[32] Kim YS (2008). Twenty-eight-day oral toxicity, genotoxicity, and gender-related tissue distribution of silver nanoparticles in sprague-dawley rats. Inhal. Toxicol..

[33] Kim YS (2010). Subchronic oral toxicity of silver nanoparticles. Part. Fibre Toxicol..

[34] Reagan-Shaw S, Nihal M, Ahmad N (2008). Dose translation from animal to human studies revisited. FASEB J. Off. Publ. Fed. Am. Soc. Exp. Biol..

[35] Jain AK (2010). Data clustering: 50 years beyond k-means. Pattern Recogn. Lett..

[36] MATLAB. *MATLAB 9.8 and Statistics Toolbox* (The MathWorks Inc., Natick, Massachusetts, 2020).

[37] Wilkin, G. A. & Huang, X. K-means clustering algorithms: implementation and comparison. In *Second International Multi-Symposiums on Computer and Computational Sciences (IMSCCS 2007)*, 133–136 (IEEE, 2007).

[38] Nidheesh N, Nazeer KA, Ameer P (2017). An enhanced deterministic k-means clustering algorithm for cancer subtype prediction from gene expression data. Comput. Biol. Med..

[39] Vouros A, Langdell S, Croucher M, Vasilaki E (2021). An empirical comparison between stochastic and deterministic centroid initialisation for k-means variations. Mach. Learn..

[40] Zappia L, Oshlack A (2018). Clustering trees: a visualization for evaluating clusterings at multiple resolutions. GigaScience.

[41] Gerecke U, Sharkey NE, Sharkey AJ (2003). Common evidence vectors for self-organized ensemble localization. Neurocomputing.

[42] Sharkey, A. & Sharkey, N. Diversity, selection, and ensembles of artificial neural nets. *Neural Networks and their Applications (NEURAP 97)* 205–212 (1997).

[43] Siegel, S. *Nonparametric Statistics for the Behavioral Sciences.* (McGraw-Hill, 1956).

