# Supplementary material for: Strategies discovery in the active allothetic place avoidance task
Source: Sci Rep. 2022 Jul 25;12:12675. doi: 10.1038/s41598-022-16374-1 (PMC9314408; doi:10.1038/s41598-022-16374-1)
Supplement: Supplementary file 1 — Supplementary Information. [file 41598_2022_16374_MOESM1_ESM.pdf]

Supplementary Material for the article "Strategies Discovery in the Active Allothetic Place Avoidance Task"

Avgoustinos Vouros   Tiago V. Gehring   Bartosz Jura
Malgorzata J. Wesierska   Daniel K. Wójcik
Eleni Vasilaki

May 13, 2022

Variation of subsegmentation threshold

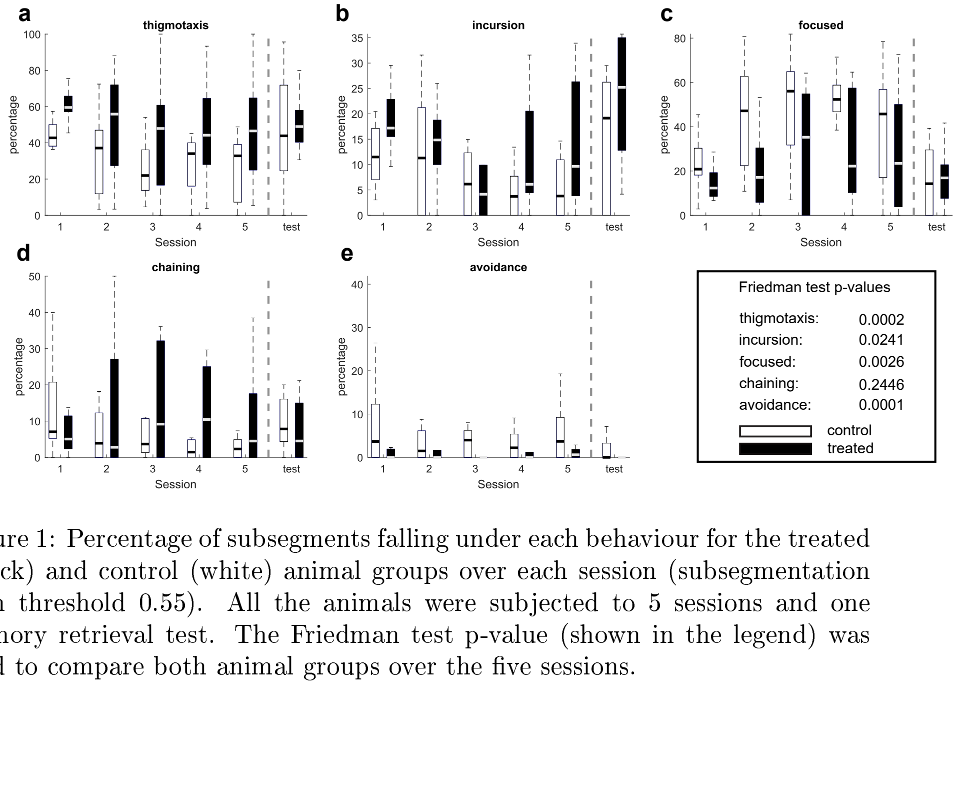

Figure 1: Percentage of subsegments falling under each behaviour for the treated (black) and control (white) animal groups over each session (subsegmentation with threshold 0.55). All the animals were subjected to 5 sessions and one memory retrieval test. The Friedman test p-value (shown in the legend) was used to compare both animal groups over the five sessions.

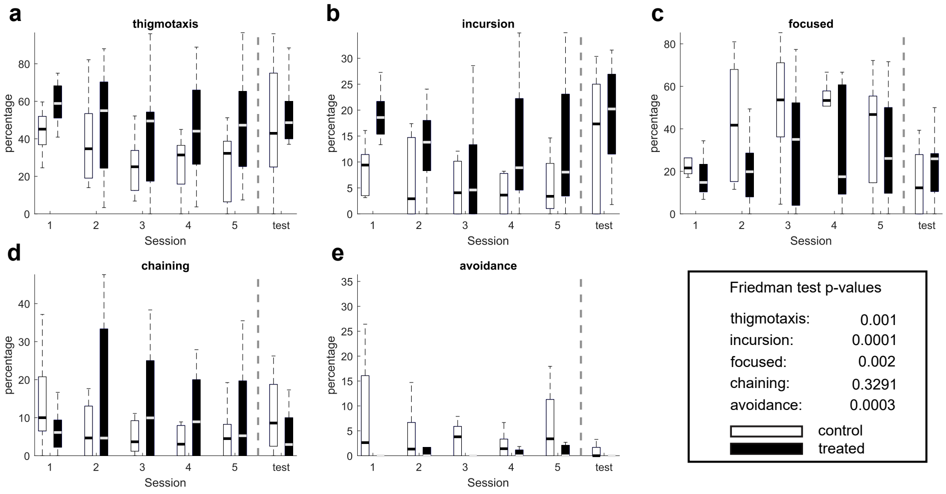

Figure 2: Percentage of subsegments falling under each behaviour for the treated (black) and control (white) animal groups over each session (subsegmentation with threshold 0.5). All the animals were subjected to 5 sessions and one memory retrieval test. The Friedman test p-value (shown in the legend) was used to compare both animal groups over the five sessions.

Clustering Visualisation

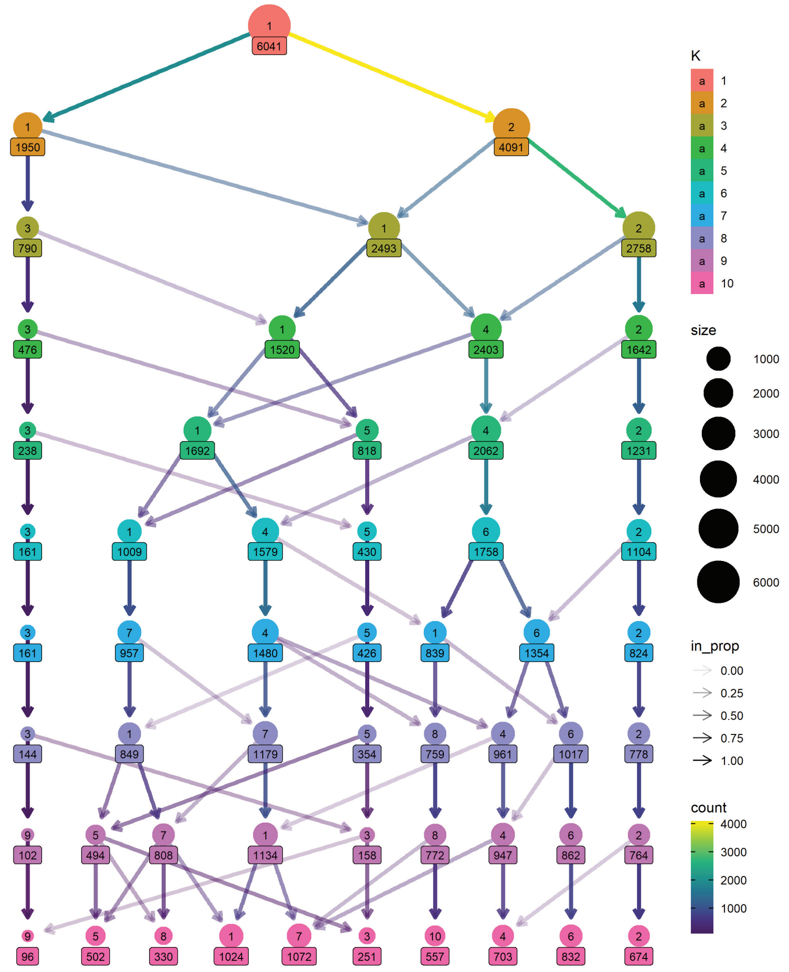

Figure 3: The R package *clustree* was used to visualise how samples move as the number of clusters increases from 1 to 10.
